# Objective and subjective cognitive status after intensive care unit treatment for COVID-19

**DOI:** 10.1016/j.bbih.2024.100786

**Published:** 2024-05-06

**Authors:** Kristina Struksnes Fjone, Jan Stubberud, Eirik Alnes Buanes, Milada Hagen, Jon Henrik Laake, Kristin Hofsø

**Affiliations:** a Department of Research and Development, Division of Emergencies and Critical Care, Oslo University Hospital, Oslo, Norway; b Department of Public Health, Oslo Metropolitan University, Oslo, Norway; c Section for Physiotherapy, Department of Clinical Services, Division of Cancer Medicine, Oslo University Hospital, Oslo, Norway; d Department of Psychology, University of Oslo, Oslo, Norway; e Department of Research, Lovisenberg Diaconal Hospital, Oslo, Norway; f Norwegian Intensive Care and Pandemic Registry, Haukeland University Hospital, Bergen, Norway; g Department of Anaesthesia and Intensive Care Medicine, Division of Emergencies and Critical Care, Oslo University Hospital, Oslo, Norway; h Department of Postoperative and Intensive Care Nursing, Division of Emergencies and Critical Care, Oslo University Hospital, Oslo, Norway; i Lovisenberg Diaconal University College, Oslo, Norway

**Keywords:** Cognitive impairment, COVID-19, Intensive care unit, Post-COVID condition, Cognitive complaints, Long-term outcome

## Abstract

**Purpose:**

Intensive care unit (ICU) survivors can experience wide-ranging and long-lasting symptoms after hospital discharge. Cognitive impairment has received increased attention in relation to the COVID-19 pandemic and can affect patients’ long-term quality of life. This study aimed to investigate the prevalence of cognitive impairment using an objective neurocognitive test 6 and 12 months following ICU admission and possible predictive factors for scoring below the defined cut-off. We also explored the prevalence of subjective cognitive complaints at 12 months, including the associated factors.

**Methods:**

This was a prospective observational study of a national cohort of COVID-19 ICU survivors during the three first pandemic waves in Norway. Data was collected by the Norwegian Intensive Care and Pandemic Registry and the study group.

**Results:**

At the six-month follow-up, 23.1% (95% CI [18.2─28.5]) of the 273 respondents scored below the cut-off on the Mini-MoCA, indicating mild cognitive impairment. At the 12-month follow-up, the prevalence declined to 11.1% (95% CI [7.5─15.6]) in 253 respondents. Older age (OR 1.06, 95% CI [1.02─1.12]) and depression (OR 1.25, 95% CI [1.07─1.55]) were associated with cognitive impairment at six months. At 12 months, almost half of the patients reported subjective cognitive complaints. Symptoms of mental health problems and fatigue were associated with subjective cognitive complaints in our exploratory analyses.

**Conclusion:**

Cognitive impairment declined significantly from 6 to 12 months in this cohort of COVID-19 ICU patients, while subjective cognitive complaints remained high at 12 months, perhaps attributed to a high total symptom burden.

## Highlights

• Cognitive impairment was measured with the Mini-MoCA at 6 and 12 months after ICU admission in a cohort of Norwegian COVID-19 survivors.
• Cognitive impairment was found to improve from 6 to 12 months after ICU admission when using a neurocognitive test.
• Older age and having symptoms of depression were associated with cognitive impairment at 6 months following ICU admission.
• Subjective cognitive complaints were measured at 12 months after ICU admission.
• When measuring subjective cognitive complaints after 12 months, a high number of patients reported complaints, such as difficulty concentrating and poor memory.

## 1 Introduction

The SARS-CoV-2 pandemic (COVID-19) has increased awareness of long-lasting symptoms following critical illness, with numerous studies reporting a high prevalence of symptoms in survivors of severe COVID-19 (Nakanishi et al., 2021). Complex symptoms in intensive care unit (ICU) survivors had been well documented through the term post-intensive care syndrome (PICS) long before the COVID-19 pandemic (Elliott et al., 2014). PICS conceptualises new or worsening symptoms of physical, psychological, and cognitive health arising after critical illness and persists beyond acute hospitalisation (Elliott et al., 2014). The pre-pandemic literature consistently highlighted cognitive impairment as a common long-term symptom in ICU patients, especially those with acute respiratory failure (ARF) and acute respiratory distress syndrome (ARDS) (Honarmand et al., 2020; Mikkelsen et al., 2012; Pandharipande et al., 2013). As ARF and ARDS are the primary reasons for ICU admission in COVID-19 (Tan et al., 2021), cognitive impairment in this population of ICU survivors has garnered much attention (Schou et al., 2021). Typical cognitive challenges after ICU treatment include difficulties with concentration, memory, and executive functions (e.g. verbal fluency), all of which can significantly impact quality of life, daily functioning, and return to work (Honarmand et al., 2020; Mikkelsen et al., 2012). A recent systematic review of psychiatric and neurocognitive sequela of COVID-19 identified that research in this area has been characterised by limited sample sizes, inconsistent assessment methods, and heterogeneous patient populations (Schou et al., 2021). Noteworthy, few studies have included both subjective (e.g. rating scale) and objective (e.g. performance-based neurocognitive test) cognition measures in studies with COVID-19 ICU patients, and there is a knowledge gap concerning the agreement between these measurement methods (Costas-Carrera et al., 2022; Godoy-González et al., 2023; Pihlaja et al., 2023).

Thus, the present study aimed to investigate both objective cognitive impairment and subjective cognitive complaints in a national cohort of ICU survivors of COVID-19. The objectives were to 1) estimate the prevalence of cognitive impairment at 6 and 12 months following ICU admission due to COVID-19; 2) identify possible predictive factors associated with objective long-term cognitive impairment at 6 and 12 months in survivors of ICU treatment due to COVID-19; 3) compare the prevalence of objective cognitive impairment with subjective cognitive complaints at 12 months following ICU admission due to COVID-19; and 4) explore the possible predictive factors associated with subjective cognitive complaints at 12 months following ICU admission.

## 2 Materials and methods

### 2.1 Study design and setting

The present study is a registry-based prospective observational study of COVID-19 patients admitted to ICUs in Norway. It was conducted as a collaboration between Oslo University Hospital and the Norwegian Intensive Care and Pandemic Registry (NIPaR), which serves as a national registry of all ICU patients and patients with COVID-19 admitted to hospital, where registration is mandatory. Additional information regarding the NIPaR can be found in a separate publication (Fjone et al., 2023). Our report adheres to the Strengthening the Reporting of Observational Studies in Epidemiology (STROBE) guidelines for comprehensive and transparent reporting of observational studies (Supplementary File 1) (von Elm et al., 2007).

### 2.2 Study population

The study included ICU survivors aged 18 years and above, who were diagnosed with COVID-19 through polymerase chain reaction (PCR) testing. Eligible participants were those admitted to any Norwegian ICU between March 1, 2020, and June 30, 2021, registered in the NIPaR database, and completed the neurocognitive test at the six-month follow-up. To be included in the NIPaR dataset the ICU needs to fulfil all the following criteria: 1) have a defined area for ICU treatment; 2) be equipped and monitored for ICU; 3) have ICU-educated nurses and doctors and 4) manage ICU patients on a daily basis. From these units the following patients are registered in the NIPaR when at least one of the criteria are fulfilled: 1) stays >24 hours; 2) stays with ventilatory support; 3) deaths <24 hours; 4) transferals to other ICU <24 hours and/or 5) stays with continuous infusion of vasoactive substances. Patients with a lack of proficiency in Norwegian were excluded from the study.

### 2.3 Outcomes

The outcomes of the present study were objective cognitive impairment, assessed with the Mini Montreal Cognitive Assessment (Mini-MoCA) version 2.1 (Wong et al., 2015), and self-reported cognitive complaints, measured with four items addressing cognitive function from the Chalder Fatigue Scale (CFQ) (Cella and Chalder, 2010).

### 2.4 Data collection

The NIPaR identified the patients and collected clinical and demographic data from the participating ICUs. Follow-up data were collected both from the registry and by the study group (see Supplementary File 2 for details). The NIPaR collected data through electronic resources (Helsenorge or Digipost) or by mail. The study group collected additional patient-reported outcome measures (PROMs) in conjunction with the telephone-administrated neurocognitive test. One certified researcher (first author) conducted all Mini-MoCA assessments as part of structured telephone interviews. To avoid language bias, the patients’ language skills were evaluated by the researcher, and those not proficient in Norwegian were excluded from the Mini-MoCA.

### 2.5 Clinical and demographic data

The clinical data included predefined risk factors associated with severe COVID-19 (e.g. chronic disease, smoking status, etc.), a severity of illness measure (Simplified Acute Physiology Score (SAPS II) (Le Gall et al., 1993)), ICU therapies (e.g. mechanical ventilation (MV), extracorporeal membrane oxygenation (ECMO)), ICU length of stay (LOS), frailty score (i.e. Clinical Frailty Scale (Rockwood et al., 2005)), peripheral oxygen saturation, and respiratory rate at hospital admission. The demographic data included gender, age at ICU admission, and information regarding co-habitation, employment, and level of education (collected six months after post ICU-admission).

### 2.6 The Mini Montreal Cognitive assessment

The Mini-MoCA is recommended for cognitive impairment screening in ARF survivors (Wong et al., 2015; Needham, 2020). This neurocognitive test was translated into Norwegian following standard forward−backward procedures (Wild et al., 2005) and approved for use by MoCA Inc© without psychometric testing. The results of the Mini-MoCA were used to define objective cognitive impairment in the present study. It consists of four subtests covering five cognitive domains: attention, verbal learning and memory, executive function (verbal fluency), and orientation (Wong et al., 2015). Attention, verbal learning, and memory are measured with subtest 1 and subtest 3 of both immediate and delayed recall of five words, respectively (Wong et al., 2015). Executive function (verbal fluency) is assessed in subtest 2, naming as many words as possible beginning with a certain letter in 1 minute. The subject needs to name 14 words or more to achieve the highest score of 4 points. Lastly, the ability to orient oneself according to both time and place is assessed (Wong et al., 2015). The overall score ranges from 0 to 15, and in the present study, cognitive impairment was defined as a total score <11 (Nasreddine, 2020). In the instruction manual, this is the recommended cut-off from the developers of the neurocognitive test (Nasreddine, 2020).

### 2.7 The Chalder Fatigue Scale

The CFQ was originally designed to measure fatigue and contains 11 items (seven for physical fatigue and four for mental (cognitive) fatigue) (Cella and Chalder, 2010) and has been validated for the Norwegian population, with available norm data (Loge et al., 1998). We measured self-reported cognitive complaints using four CFQ items: “Do you have difficulties concentrating?” “Do you make slips of the tongue when speaking?” “Do you find it more difficult to find the correct word?” “How is your memory?” Each of the items has a global scoring range from 0 (less than usual) to 3 (much more than usual), except for the last question regarding memory, where the score ranges from 0 (better than usual) to 3 (much worse than usual) (Cella and Chalder, 2010). The total score from the CFQ can be used as either a continuous or a bimodal score. To our knowledge, there is no consensus regarding a cut-off score for the continuous scale, but when using bimodal scoring, one can define a case of fatigue with a score of 4 or above which was used for the present study (Cella and Chalder, 2010). We used bimodal scoring, categorising the two lowest as 0 and the two highest as 1, to describe prevalence and to perform logistic regression analyses at the 12 months follow-up (Cella and Chalder, 2010). Additionally, we investigated the complete CFQ, providing ordinal fatigue data as a possible predictive factor for the primary outcome at 12 months and we used item 1 (“Do you have problems with tiredness”) as a covariate in our exploratory analyses with subjective cognitive complaints as the dependent variable.

### 2.8 Self-reported covariates

Symptoms of anxiety and depression were measured with the Hospital Anxiety and Depression Scale (HADS) (Snaith, 2003); symptoms of post-traumatic stress (PTSS) were measured with the Impact of Event Scale-6 (IES-6) (Hosey et al., 2019); and self-perceived dyspnea was measured with the modified Medical Research Council Dyspnea Scale (mMRC) (Cotes, 1987). We assessed rehabilitation using a questionnaire developed by the study group, which covered both ICU- and post-ICU rehabilitation. Total scores were used for all the questionnaires, except for the rehabilitation questionnaire, when used as covariates. Further details on each questionnaire are provided in Supplementary File 2.

### 2.9 Statistical analyses

The descriptive data are presented as frequencies (counts) and proportions (percentages), and the continuous variables are presented as medians and ranges (min−max). To compare the respondents with non-respondents, the Mann−Whitney *U* test was used for variables with skewed distributions, and the independent *t*-test was used for normally distributed variables. Point estimates of prevalence are presented with 95% confidence intervals (CI), derived using binomial approximation. The strength of the possible associations between Mini-MoCA and selected possible predictive factors were assessed using logistic regression models. We included covariates based on both clinical (i.e. respiration rate, peripheral oxygen saturation, ECMO) and empirical (i.e. age, gender, predefined risk factors, SAPS II score, Clinical Frailty Scale, ICU LOS, duration of MV, HADS scores, CFQ total score, mMRC score) considerations. Variables with a *p*-value of 0.1 or less from the univariate analyses were included in the multiple logistic regression models, and all models were adjusted for gender. We used backward stepwise logistic regression to explore the association between self-reported subjective cognitive complaints and possible predictive factors. We used the same covariates as in the regression models for the Mini-MoCA but excluded the pre-defined risk factors, time on MV, respiratory rate, and mMRC to avoid overfitting and due to the lack of a clinical rationale for their inclusion. Additionally, we included item 1 from the CFQ as a measure of overall fatigue to explore the association between subjective cognitive complaints and fatigue. To estimate the CIs for the regression coefficients, we employed bootstrapping with 1000 repetitions. All tests were two-sided, and we considered *p*-values below 0.05 as statistically significant. All analyses were considered exploratory, so no correction for multiple testing was done. The statistical analyses were performed in IBM SPSS Statistics (version 29) and Stata (version 17.0).

### 2.10 Ethics

The study was approved by the Regional Committees for Medical and Health Research Ethics (reference number: 135310) and the institutional data protection officer. The data collection by the NIPaR was conducted with a written consent waiver (national medical quality registry). Patients or their proxies were informed about the registry during and after the ICU stay, clarifying their reservation rights, including the option to have their data deleted at any time. Written consent was obtained for all patients participating in the telephone interviews and/or completing the questionnaires from the study group.

## 3 Results

### 3.1 Patient characteristics

**Fig. 1:**
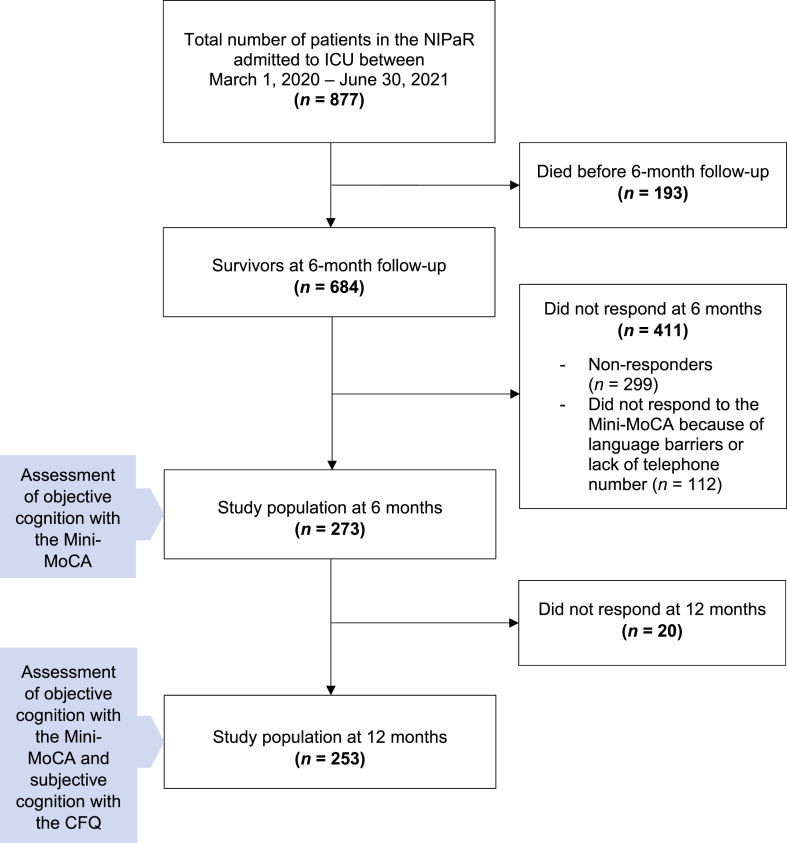
Flowchart.

During the period March 1, 2020, and June 30, 2021, 877 patients with COVID-19 were admitted to Norwegian ICUs, 193 of whom died before six-month follow-up leaving, 684 patients eligible for neurocognitive testing. Altogether, 273 patients responded to the Mini-MoCA assessment at six months (Fig. 1 Flowchart). Table 1 presents the clinical characteristics at ICU admission and the sociodemographic characteristics measured at six months after ICU admission. The median age of study participants was 60 (18−88) years, and the majority were male (70.3%). The median ICU LOS was 11.1 (0.5−76.2) days, and 87.5% received MV (Table 1). Comparisons between the respondents and non-respondents at six months are shown in Supplementary File 2: Table 2. The respondents were older at ICU admission, had a higher SAPS II score, and stayed longer in the ICU and on MV than the non-respondents.

**Table 1:** Characteristics of the study population (*n* = 273).

Clinical characteristics at time of ICU admission	*n*	%	Median (range)
**Age**			60 (18─88)
**Gender**			
Female	81	29.7	
Male	192	70.3	
**Clinical Frailty Scale**	185		2 (1─7)
**Peripheral oxygen-saturation** (%)	246		90 (47─100)
**Respiration rate** (per minute)	265		26 (8─78)
**SAPS II score**	273		31 (6─72)
**ICU length of stay** (days)	273		11.1 (0.5─76.2)
**Received mechanical ventilation**	239	87.5	
**Time on mechanical ventilation** (days)			9.1 (0.1─69.7)
**Type of mechanical ventilation**			
Time on invasive mechanical ventilation	156		12.3 (0.8─69.5)
Time on non-invasive ventilation	173		1.3 (0.1─11.7)
**Received ECMO**	5	1.8	
**Any risk factor**			
Yes	226	82.8	
No	47	17.2	
**Risk factors**^a^			
Cardiovascular disease	111	40.7	
Obesity	76	27.8	
Asthma	53	19.4	
Diabetes Mellitus I or II	40	14.7	
Chronic lung disease (asthma not included)	26	9.5	
Immune deficit	17	6.2	
Kidney disease	15	5.5	
Cancer	12	4.4	
Neurological disease	5	2.9	
Smoker	8	2.9	
Liver disease	1	0.4	
Pregnancy	1	0.4	
**Sociodemographic characteristics measured at six months**	***n***	%	
**Co-habitation** (*n* = 210)			
Living with someone	175	83.3	
Living alone	35	16.7	
**Educational status** (*n* = 212)			
Primary/secondary school	112	53	
Higher education – College/university	100	47	
**Mental health symptoms**			
Anxiety ≥8 (*n* = 270)	57	21.1	
Depression ≥8 (*n* = 272)	50	18.4	
PTSS ≥1.75 (*n* = 208)	56	26.9	

a some have more than one risk factor.

### 3.2 Prevalence of objective cognitive impairment

Six months following ICU admission, 23.1% (95% CI [18.2−28.5]) of the 273 patients who completed the neurocognitive testing scored below the cut-off value (<11) on the Mini-MoCA, indicating cognitive impairment. At 12 months, the prevalence of cognitive impairment in 253 respondents was 11.1% (95% CI [7.5−15.6]).

### 3.3 Factors associated with objective cognitive impairment

In the logistic regression model for the Mini-MoCA assessed at the six-month follow-up, we found that older age (OR 1.06, 95% CI [1.02−1.12]) and having symptoms of depression (OR 1.25, 95% CI [1.07−1.55]) were significantly associated with scoring below 11 on the Mini-MoCA (Table 2). At 12 months, only 28 patients scored below the cut-off value on the Mini-MoCA. In the multivariate analyses, older age (OR 1.09, 95% CI [1.02−1.16]) and a higher score in the Clinical Frailty Scale (OR 1.61, 95% CI [1.01−2.57]) were statistically significantly associated with scoring below the cut-off score of 11. Details regarding the 12-month logistic regression model are available in Supplementary File 2: Table 3.

**Table 2:** Logistic regression analyses. Predictive factors associated with the Mini-MoCA score <11 at six months following ICU admission (*n* = 273).

	Univariate analyses			Multivariate analyses		
	OR	95% CI	*p-*value	OR	95% CI	*p-*value
**During admission**						
**Age**	1.02	0.99─1.05	**0.06**	1.06	1.02─1.12	**<0.01**
**Gender** (ref. male)	1.37	0.75─2.50	0.29	1.7	0.74─3.81	0.20
**Any risk factor yes/no** (ref. no)	1.22	0.63─2.35	0.55			
**Number of risk factors**	0.75	0.82─1.42	0.60			
**Cancer**	0.11	0.30─4.26	0.83			
**Immune deficit**	−0.03	0.31─3.10	0.94			
**Diabetes**	−0.42	0.31─1.38	0.26			
**Cardiovascular disease**	−0.12	0.50─1.57	0.69			
**Obesity**	0.16	0.62─2.23	0.62			
**Asthma**	0.17	0.57─2.46	0.65			
**Chronic lung disease (asthma not included)**	−0.44	0.27─1.56	0.33			
**Kidney disease**	0.19	0.33─4.44	0.77			
**Liver disease**	a	a	a			
**Neurological disease**	a	a	a			
**Pregnancy**	a	a	a			
**Smoker**	a	a	a			
**SAPS II Score**	1.01	0.99─1.04	0.28			
**Clinical Frailty Scale**	0.92	0.79─1.08	0.31			
**ICU LOS** (days)	0.99	0.99─1.01	0.81			
**Duration of MV** (hours)	1.00	0.99─1.00	0.69			
**Peripheral oxygen-saturation**	1.02	0.98─1.05	0.35			
**Respiration rate** (per minute)	1.01	0.98─1.05	0.26			
**ECMO**	b	b	b			
***At six months***						
**HADS anxiety** (sum score)	1.06	0.99─1.12	**0.06**	0.92	0.77─1.07	0.29
**HADS depression** (sum score)	1.12	1.05─1.19	**<0.001**	1.25	1.07─1.55	**<0.01**
**IES-6** (sum score)	1.07	1.01─1.13	**0.03**	1.04	0.93─1.16	0.43
**Received rehabilitation after hospital discharge** (ref. no)	1.50	0.76─2.83	0.21			
**mMRC**	1.23	0.89–1.69	0.20			

*CI:* Confidence interval; *ECMO:* Extracorporeal membrane oxygenation; *HADS*: Hospital Anxiety and Depression Scale; *ICU LOS:* intensive care unit length of stay; *mMRC:* Modified Research Council Dyspnea Scale; *MV:* mechanical ventilation; *OR:* Odds ratio; *SAPS:* Simplified Acute Physiology Score II. Level of significance <0.05. The Mini-MoCA score was used as a dichotomous dependent variable.

a The model could not generate results due to the low number of patients with these risk factors.

b The model could not generate results due to the low number of patients receiving extracorporeal membrane oxygenation.

### 3.4 Prevalence of self-reported cognitive complaints and associated factors

**Fig. 2:**
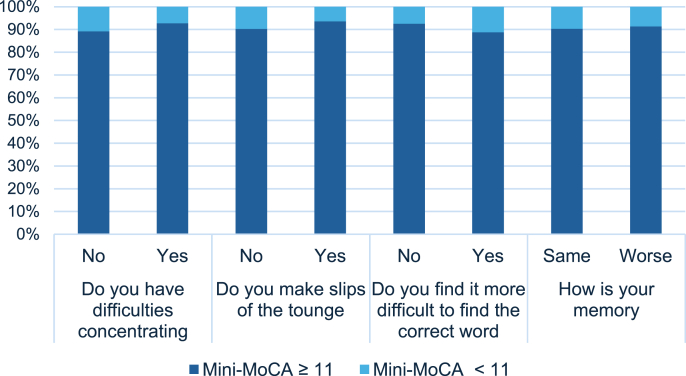
Comparison between subjective cognitive complaints and objective cognitive impairment assessment at 12 months following ICU admission The figure represents patients that have responded to both Mini-MoCA and The Chalder Fatigue Scale at 12 months follow-up and underlies that regardless of the results of the neurocognitive test, the distribution of patients reporting subjective cognitive complaints is almost the same.

Among the 174 patients who responded to both the CFQ and Mini-MoCA at 12 months, 16 (9.2%) scored below the cut-off value of 11 on the Mini-MoCA; 84 (47.1%) reported that they had more or much more difficulty concentrating; 31 (17.8%) reported that they experienced more or many more slips of the tongue; and 80 (46%) had more or much more difficulty finding the correct word. Eighty-one (46.6%) patients reported that their memory was worse or much worse than usual. The prevalence of the CFQ items in patients scoring over and below the cut-off on the Mini-MoCA is presented in Fig. 2 (Comparison between subjective cognitive complaints and objective cognitive impairment assessment at 12 months following ICU admission). In the explorative analyses (Table 3), we identified an association of symptoms between posttraumatic stress (OR 1.2, 95% CI [1.13−1.28]) and feeling tired (OR 3.81, 95% CI [2.38−6.09]) with difficulty concentrating. Depressive symptoms (OR 1.27, 95% CI [1.16−1.39]) were associated with more slips of the tongue, and feeling tired was associated with greater difficulty finding the correct word (OR 4.94, 95% CI [2.67−9.13]) and experiencing worse memory than usual (OR 4.25, 95% CI [2.90−6.22]). Step 1 for each item in the backward stepwise logistic regression model are presented in Supplementary File 2: Table 4.

**Table 3:** Multivariate logistic regression of subjective cognitive complaints on selected covariates^a^ (*n* = 174).

	Do you have difficulties concentring			Do you make slips of the tongue			Do you find it more difficult to find the correct word			How is your memory		
	OR	95% CI	*p-* value	OR	95% CI	*p-*value	OR	95% CI	*p-*value	OR	95% CI	*p-*value
**IES-6**	1.2	1.13─1.28	<0.01									
**HADS depression**				1.27	1.16─1.39	<0.01						
**CFQ item 1**	3.81	2.38─6.09	<0.01				4.94	2.67─9.13	<0.01	4.25	2.90─6.22	<0.01

*CFQ item 1: The* Chalder Fatigue Scale “Do you have problems with tiredness”; *IES-6*: The Impact of Event Scale-6; *HADS:* The Hospital Anxiety and Depression Scale; *OR:* Odds ratio. Level of significance <0.05.

a Adjusted for age, gender, Clinical Frailty Scale score, SAPS II score, intensive care unit length of stay, oxygen saturation at admission, the Hospital Anxiety and Depression anxiety total score at 12 months.

## 4 Discussion

In this national observational study of Norwegian ICU patients with COVID-19, 23.1% of the 273 respondents showed cognitive impairment on a performance-based neurocognitive test (Mini-MoCA) six months after ICU admission. This prevalence declined to 11.1% at the 12-month follow-up. Conversely, the prevalence of self-reported cognitive complaints was high at the 12-month follow-up, with approximately half of the patients reporting a negative change in concentration, word finding, and memory. This result substantiates the disparity between objective and subjective measured cognition shown in previous studies involving ICU and non-ICU patients (Zlatar et al., 2017; Blackmon et al., 2022). For example, the correlations between self-reports and performance-based cognitive measures tend to be weak to moderate in patients with depression, who often report more subjective cognitive complaints than what is revealed in performance-based measures (Serra-Blasco et al., 2019; Douglas et al., 2018). Our exploratory analyses identified an association between symptoms of depression and posttraumatic stress and subjective cognitive complaints, supporting previous findings regarding COVID-19 patients (Zlatar et al., 2017; Liu et al., 2023). Furthermore, depressive symptoms were found to be associated with scoring below the Mini-MoCA cut-off at six months. The association between depression and cognition was described in a recent study with 519 ICU patients, which also found an association between early post-ICU depression and long-term cognitive impairment at 12 months (Nordness et al., 2021). A systematic review also described depression as manifesting with cognitive deficits across several domains (Semkovska et al., 2019). We did not, however, detect this association in our 12-month regression analysis (with the neurocognitive test as the dependent variable), which we believe is mainly due to the low number of patients scoring below the cut-off value on the Mini-MoCA at that point. We argue that assessing objective cognitive functioning (e.g. efficiency of cognitive processes in a structured setting) and subjective cognitive functioning (e.g. mastery of daily activities) represents two distinct constructs and that subjective measures should not be overlooked even if objective results fall within the normal range. These findings emphasise the importance of also incorporating a subjective measure of cognition into the follow-up of ICU survivors after COVID-19.

We found no significant association between fatigue and objective cognitive impairment 12 months after ICU admission, perhaps due to the low number of patients scoring below the cut-off on the Mini-MoCA. However, we identified an association between fatigue and subjective cognitive complaints, warranting further investigation of the relationship between fatigue and subjective and objective cognitive functioning. As the association between fatigue and cognitive impairment has been described in other patient populations, such as cancer and multiple sclerosis (Menzies et al., 2021), and both symptoms have received increased attention in relation to the COVID-19 pandemic (Ceban et al., 2022), this should be examined in future studies. We further speculate whether the high number of subjective cognitive complaints can be explained by a high symptom burden in these patients, which has been widely described in COVID-19 patients in particular (Lopez-Leon et al., 2021; Yang et al., 2022) and ICU survivors in general (Herridge and Azoulay, 2023). Co-occurring symptoms are also known to enhance the experience of individual symptoms and negatively affect various performance outcomes (e.g. cognitive functioning), as described in the theory of unpleasant symptoms (Lenz and Pugh, 2003).

Last, we found older age to be associated with objectively assessed cognitive impairment at both 6- and 12-months following ICU admission due to COVID-19. Age is a well-established risk factor for cognitive impairment, particularly after critical illness (Collet et al., 2021; Murman, 2015). A study assessing cognition in 237 ICU survivors before the pandemic found that older age was the only statistically significant risk factor for poorer neurocognitive status six months after ICU admission (Collet et al., 2021), a result corroborated in a recent study of COVID-19 ICU patients (Godoy-González et al., 2023). We investigated the potential for nonresponse bias by comparing the demographic and clinical data relating to the respondents and non-respondents. The respondent group was older, more severely ill, and spent more time in the ICU and on MV than patients who did not respond to the Mini-MoCA at six months. One might therefore anticipate a high prevalence of cognitive impairment in the respondent group. However, our cohort exhibited a comparatively low prevalence of cognitive impairment at both time points, and we consider the likelihood of overestimating the magnitude of cognitive impairments as low. It is also noteworthy that our study population had a relatively low median age, which could be one of the explanations for the low prevalence of cognitive impairment in the present study.

### 4.1 Limitations

There are some limitations to our study. First, the use of the Mini-MoCA and cut-offs to measure cognitive impairment might be questioned (Ihle-Hansen et al., 2017). The MoCA and its shortened version for telephone use are recommended screening tools for ARF survivors (Needham et al., 2017), with high sensitivity and specificity in identifying mild cognitive impairment (Nasreddine et al., 2005; McDicken et al., 2019). While, Mini-MoCA's primary advantage lies in its brevity, it has not been validated for ICU populations, a noteworthy methodological consideration. Furthermore, practice effects cannot be ruled out since the same version of the Mini-MoCA was used at both measurement points but we consider this as unlikely since it was six months between the assessments.

Second, the CFQ items used to measure subjective cognitive complaints lacked formal validation for this purpose, although prior research has utilised them for assessing subjective cognition (Fjelltveit et al., 2022). The four selected CFQ items offer the advantage of enabling a comparison of cognitive complaints with the patients' premorbid status, as they provided response categories that allowed the patients to express changes in subjective cognitive functioning. This offers insight into patients’ perceptions of cognitive change and facilitates change detection.

Third, only participants fluent in Norwegian completed the Mini-MoCA, which may have biased our patient sample towards ethnic homogeneity and may also have contributed to a lower response rate. Due to time constraints, the Mini-MoCA was not translated into other languages, limiting generalisability, especially considering the significant number of patients of non-Norwegian origin during the initial COVID-19 waves in Norway (Indseth et al., 2021). Another limitation is that we did not control for or describe the different variants of the virus in our statistical models.

Lastly, delirium is a recognised risk factor for developing long-term cognitive impairment and should have been considered as a covariate (Girard et al., 2010; Goldberg et al., 2020; Lee et al., 2020). Due to limitations in the NIPaR dataset, data on ICU delirium were not available for the present study.

## 5 Conclusions

In this national cohort of COVID-19 ICU patients, there was a significant decline in objective cognitive impairment from 6 to 12 months following ICU-admission. Interestingly, subjective cognitive complaints were significantly more prevalent than objective cognitive impairment at 12 months, with mental health problems and fatigue showing associations with these complaints in our exploratory analyses. These findings could be attributed to a high overall symptom burden. Our results should be replicated in larger datasets with age and sex matched controls and include a more comprehensive neurocognitive test battery, a validated measure of subjective cognition, and a formal assessment of premorbid cognitive (dys)function.

## Ethics approval

This study was performed in line with the principles of the Declaration of Helsinki. The study was approved the Regional Committees for Medical and Health Research Ethics (reference number: 135310) and by the institutional privacy representative.

## Consent

Informed consent was obtained from all participants included in the study.

## Funding

This work was funded by the 10.13039/501100005416Research Council of Norway [grant numbers: 312712/CR, 2020].

## CRediT authorship contribution statement

**Kristina Struksnes Fjone:** Writing – review & editing, Writing – original draft, Visualization, Validation, Methodology, Investigation, Formal analysis, Data curation. **Jan Stubberud:** Writing – review & editing, Writing – original draft, Validation, Supervision, Methodology. **Eirik Alnes Buanes:** Writing – review & editing, Writing – original draft, Visualization, Validation, Supervision, Project administration, Methodology, Funding acquisition, Formal analysis, Data curation, Conceptualization. **Milada Hagen:** Writing – review & editing, Visualization, Validation, Supervision, Methodology, Investigation, Formal analysis, Data curation, Conceptualization. **Jon Henrik Laake:** Writing – review & editing, Visualization, Validation, Software, Methodology, Funding acquisition, Formal analysis, Conceptualization. **Kristin Hofsø:** Writing – review & editing, Writing – original draft, Visualization, Validation, Supervision, Project administration, Methodology, Investigation, Funding acquisition, Formal analysis, Data curation, Conceptualization.

## Declaration of competing interest

The authors declare the following financial interests/personal relationships which may be considered as potential competing interests:

Kristin Hofso reports financial support was provided by 10.13039/501100005416Research Council of Norway. The authors declare that they have no known competing financial interests or personal relationships that could have appeared to influence the work reported in this paper.

## Data Availability

Data will be made available on request.
