# Supplementary material for: Objective and subjective cognitive status after intensive care unit treatment for COVID-19
Source: Brain Behav Immun Health. 2024 May 6;38:100786. doi: 10.1016/j.bbih.2024.100786 (PMC11103414; doi:10.1016/j.bbih.2024.100786)
Supplement: Multimedia component 1 [file mmc1.docx]

**Supplementary File 2**

This supplementary file provides detailed information about the data collection and the research instruments used in the present study. In addition, we present supplementary results on the differences between the respondents and non-respondents as well as results from the univariate and multivariate logistic regression, with the Mini-MoCA as the dependent variable at the 12-month follow-up. Last, we provide details of step 1 of each item in the CFQ in the backward stepwise logistic regression model.

**METHODS SUPPLEMENT**

**Data collection**

The registry collected patient-reported outcome measures (PROMs) at 3, 6, and 12 months after ICU admission, but did not collect the same PROMs at each measurement time point. Table 1 presents an overview of measurement time points for each questionnaire. Demographic variables, for example, were only collected at the six-month follow-up, and the Chalder Fatigue Scale (CFQ) was collected at 3 and 12 months. The study group also performed telephone interviews at 6 and 12 months after ICU admission to collect data regarding rehabilitation, anxiety, depression, and objective cognition. Patients with unknown telephone numbers received the questionnaires by mail, along with a prepaid envelope and a request to write their telephone numbers on the consent form to enable neurocognitive testing by phone. The patients received a reminder by mail after one month, both from the study group and the registry.

**Table 1** Overview of data collection measurement time points

| **Measurement time point** | **Instrument** | **Collected**  **by** |
| --- | --- | --- |
| **3 months** | CFQ (subjective cognition) | The NIPaR |
| **6 months** | Mini-MoCA (objective cognition)  IES-6  HADS  Rehabilitation  mMRC  Demographic data | Study group  The NIPaR  Study group  Study group  The NIPaR  The NIPaR |
| **12 months** | Mini-MoCA (objective cognition)  IES-6  HADS  Rehabilitation  mMRC  CFQ (subjective cognition) | Study group  The NIPaR  Study group  Study group  The NIPaR  The NIPaR |

*_CFQ_* _– Chalder Fatigue Scale;_ *_HADS_* _– the Hospital Anxiety and Depression Scale;_ *_IES-6_* _– The Impact of Event Scale-6;_ *_Mini-MoCA_* _– the Mini Montreal Cognitive Assessment;_ *_mMRC_* _–the modified Medical Research Council Dyspnea Scale;_ *_The NIPaR_* _─ the Norwegian Intensive Care and Pandemic Registry_

**Self-reported covariates**

**The Hospital Anxiety and Depression Scale**

Anxiety and depression were measured with the Hospital Anxiety and Depression Scale (HADS), which comprises seven items for anxiety and seven items for depression, yielding two separate scores [2]. Each item is scored on a Likert scale from 0 (not at all) to 3 (very much) yielding a total score between 0 and 21 [2]. A cut-off of 8 on each scale is often used to define symptoms of depression and anxiety, and the total score can further classify the severity of symptoms, 8─10 (mild), 11─14 (moderate), and 15─21 (severe) [2]. In the present study, a cut-off of 8 was used to define patients with or without symptoms of depression or anxiety. The HADS has been widely used to assess anxiety and depression in ICU patients and has been recommended for this patient population [3]. It has been translated and validated for the Norwegian population [4].

**The Impact of Event Scale-6**

Symptoms of post-traumatic stress (PTSS) were measured with the Impact of Event Scale-6 (IES-6), which consists of six items scored from 0 (not at all) to 4 (extremely), yielding a total score between 0 and 24 [5]. A cut-off of 1.75 is recommended to define symptoms of PTS [5]. The IES-6 is a validated instrument for ARF patients and has shown good psychometric properties [5]. It has been translated into Norwegian, and the full version, the Impact of Event Scale-Revised, has been validated for the Norwegian population [6, 7].

**The Modified Medical Research Council Dyspnea Scale**

Self-perceived dyspnoea was measured with the modified Medical Research Council Dyspnea Scale (mMRC) [8]. This is a unidimensional questionnaire comprising five statements relating dyspnoea to everyday activities. The patients are asked to choose one of the statements, yielding a total score from 0─4. The mMRC is not validated for ICU patients but has been used in multiple studies assessing dyspnoea in different COVID-19 populations. This questionnaire has been translated into Norwegian but has not gone through full validation for the Norwegian population

All the above-mentioned questionnaires have defined cut-off values; however, in the present study, the total scores of each questionnaire were used as a continuous independent variable in the logistic regression analyses.

Rehabilitation was assessed with a questionnaire developed by the study group. It consists of seven items asking whether patients received rehabilitation while in the ICU and after discharge, the duration of rehabilitation, and where (e.g. a full body rehabilitation institution or in a municipal setting) the rehabilitation was delivered. This instrument has not been validated through psychometric testing.

**RESULTS SUPPLEMENT**

**Table 2** Characteristics of respondents and non-respondents at six months

|  | **Respondents 6 months**  (*n =* 273) | | | **Non-respondents 6 months**  (*n =* 411) | | | ***p-*value** |
| --- | --- | --- | --- | --- | --- | --- | --- |
|  | ***n*** | **%** | **Median (range)** | ***n*** | **%** | **Median (range)** |  |
| **Age** |  |  | 61 (18─87) |  |  | 57 (24─91) | **<.01** |
| **Gender** |  |  |  |  |  |  | 0.84 |
| Male | 192 | 70.3 |  | 286 | 69.6 |  |  |
| Female | 81 | 29.7 |  | 125 | 30.4 |  |  |
| **Risk factor** |  |  |  |  |  |  | 0.19 |
| Yes | 267 | 70.1 |  | 194 | 69.3 |  |  |
| No | 114 | 29.9 |  | 86 | 30.7 |  |  |
| **SAPS II score** | 273 |  | 31 (6─72) | 411 |  | 29 (6─70) | **0.02** |
| **Clinical Frailty Scale** | 185 |  | 2 (1─7) | 221 |  | 3 (1─9) | 0.14 |
| **ICU LOS** | 273 |  | 11.1 (0.5─76.2) | 411 |  | 8.8 (0.1─74.8) | **<.01** |
| **Duration of MV** | 239 |  | 91. (0.1─69.7) | 345 |  | 6.8 (0.1─64.6) | **<.01** |

*_ICU LOS_* _─ intensive care unit length of stay;_ *_MV_* _─ mechanical ventilation;_ *_SAPS_* _─ Simplified Acute Physiology Score II_

**Factors associated with cognitive impairment at 12 months after ICU admission**

The low number of patients scoring below the cut-off value of 11 in the Mini-MoCA limited the multiple logistic regression model at the 12-month follow-up. In the univariate analyses age, the SAPS II Score and the Clinical Frailty Scale score were statistically significant. Only older age (OR 1.09, 95% CI [1.00─1.23]) and the Clinical Frailty Scale score (OR 1.61, 95% CI [1.01─2.57]) was statistically significant in the multivariate analysis. Further details are presented in Table 3.

**Table 3** Logistic regression analyses. Predictive factors associated with the Mini-MoCA score <11 at 12 months after ICU admission (*n* = 253)

|  | **Univariate analyses** | | | **Multivariate analyses** | | |
| --- | --- | --- | --- | --- | --- | --- |
|  | **OR** | **95% CI** | ***p-*value** | **OR** | **95% CI** | ***p-*value** |
| ***During admission*** |  |  |  |  |  |  |
| **Age** | 1.07 | 1.03─1.12 | **0.001** | 1.09 | 1.02─1.16 | **0.01** |
| **Gender**  (ref. male) | 2.02 | 0.90─4.50 | 0.87 |  |  |  |
| **Risk factor**  (ref. no) | * | * | ***** |  |  |  |
| **SAPS II score** | 1.04 | 1.01─1.07 | **0.04** | 0.98 | 0.92─1.04 | 0.51 |
| **Clinical Frailty Scale score** | 1.55 | 1.03─2.35 | **0.04** | 1.61 | 1.01─2.57 | **0.05** |
| **ICU LOS** (days) | 1.01 | 0.99─1.03 | 0.31 |  |  |  |
| **Duration of MV** (days) | 1.01 | 0.98─1.04 | 0.47 |  |  |  |
| **Peripheral oxygen-saturation** | 1.02 | 0.96─1.07 | 0.53 |  |  |  |
| **Respiration rate** (per minute) | 0.99 | 0.95─1.04 | 0.80 |  |  |  |
| **ECMO** | ** | ** | ** |  |  |  |
| ***At 12 months*** |  |  |  |  |  |  |
| **HADS anxiety** (sum score) | 0.96 | 0.86─1.06 | 0.40 |  |  |  |
| **HADS depression** (sum score) | 1.04 | 0.93─1.15 | 0.50 |  |  |  |
| **IES-6** (sum score) | 0.99 | 0.91─1.01 | 0.94 |  |  |  |
| **Chalder Fatigue Scale** (sum score) | 0.99 | 0.90─1.07 | 0.71 |  |  |  |
| **mMRC** | 1.04 | 0.59─1.81 | 0.88 |  |  |  |

*_CI:_* _Confidence interval;_ *_ECMO:_* _Extracorporeal membrane oxygenation;_ *_HADS_* _:Hospital Anxiety and Depression Scale;_ *_ICU LOS:_* _intensive care unit length of stay;_ *_Mini- MoCA:_* _Mini Montreal Cognitive Assessment;_ *_mMRC:_* _Modified Research Council Dyspnea Scale;_ *_MV:_* _mechanical ventilation;_ *_OR:_* _Odds ratio;_ *_SAPS:_* _Simplified Acute Physiology Score II. Level of significance ˂0.05. The Mini-MoCA score was used as a dichotomous dependent variable.*The model could not generate results due to the low number of patients, with any risk factor scoring below cut-off in the Mini-MoCA. **The model could not generate results due to the low number of patients receiving extracorporeal membrane oxygenation._

**Table 4** Multivariate backward stepwise logistic regression of subjective cognitive complaints

|  | **Do you have difficulties concentrating** | | |
| --- | --- | --- | --- |
| STEP 1 | **OR** | **95% CI** | ***p*-value** |
| Age | 0.97 | 0.93─1.01 | 0.18 |
| Gender | 1.42 | 0.50─4.01 | 0.51 |
| SAPS II score | 0.99 | 0.93─1.06 | 0.81 |
| Clinical Frailty Scale score | 0.72 | 0.42─1.22 | 0.22 |
| ICU LOS | 1.01 | 0.97─1.05 | 0.58 |
| Peripheral oxygen-saturation | 0.97 | 0.92─1.02 | 0.27 |
| HADS Anxiety total score | 0.95 | 0.78─1.16 | 0.64 |
| HADS Depression total score | 0.95 | 0.76─1.19 | 0.64 |
| IES-6 total score | 1.18 | 1.02─1.36 | 0.02 |
| CFQ item 1 | 6.38 | 2.47─16.47 | <0.001 |
|  | **Do you make slips of the tongue** | | |
| STEP 1 | **OR** | **95% CI** | ***p*-value** |
| Age | 1.01 | 0.95─1.07 | 0.79 |
| Gender | 1.38 | 0.38─5.05 | 0.62 |
| SAPS II score | 0.97 | 0.89─1.07 | 0.59 |
| Clinical Frailty Scale score | 1.46 | 0.82─2.58 | 0.19 |
| ICU LOS | 1.03 | 0.98─1.07 | 0.28 |
| Peripheral oxygen-saturation | 0.99 | 0.92─1.07 | 0.83 |
| HADS Anxiety total score | 0.97 | 0.76─1.23 | 0.81 |
| HADS Depression total score | 1.38 | 1.06─1.79 | 0.02 |
| IES-6 total score | 1.02 | 0.87─1.19 | 0.83 |
| CFQ item 1 | 2.27 | 0.75─6.88 | 0.15 |
|  | **Do you find it more difficult to find the correct word** | | |
| STEP 1 | **OR** | **95% CI** | ***p*-value** |
| Age | 1.01 | 0.97─1.05 | 0.69 |
| Gender | 0.94 | 0.38─2.33 | 0.90 |
| SAPS II score | 0.99 | 0.93─1.05 | 0.72 |
| Clinical Frailty Scale score | 0.93 | 0.60─1.42 | 0.73 |
| ICU LOS | 1.00 | 0.97─1.04 | 0.81 |
| Peripheral oxygen-saturation | 1.01 | 0.96─1.06 | 0.76 |
| HADS Anxiety total score | 0.95 | 0.79─1.13 | 0.55 |
| HADS Depression total score | 1.16 | 0.95─1.42 | 0.15 |
| IES-6 total score | 1.04 | 0.93─1.15 | 0.49 |
| CFQ item 1 | 3.51 | 1.63─7.56 | <0.01 |
|  | **How is your memory** | | |
| STEP 1 | **OR** | **95% CI** | ***p*-value** |
| Age | 1.01 | 0.97─1.05 | 0.77 |
| Gender | 0.85 | 0.33─2.19 | 0.74 |
| SAPS II score | 1.00 | 0.95─1.06 | 0.95 |
| Clinical Frailty Scale score | 0.66 | 0.42─1.04 | 0.08 |
| ICU LOS | 1.00 | 0.97─1.04 | 0.89 |
| Peripheral oxygen-saturation | 1.01 | 0.96─1.05 | 0.84 |
| HADS Anxiety total score | 0.92 | 0.76─1.10 | 0.36 |
| HADS Depression total score | 1.15 | 0.93─1.42 | 0.18 |
| IES-6 total score | 1.08 | 0.96─1.20 | 0.19 |
| CFQ item 1 | 3.76 | 1.69─8.35 | <0.01 |

*_CFQ; the Chalder Fatigue Scale «Do you have problems with tiredness”; CI:_* _Confidence interval;_ *_HADS_* _:Hospital Anxiety and Depression Scale;_ *_ICU LOS:_* _intensive care unit length of stay;_ *_IES-6: the Impact of Event Scale-6; OR:_* _Odds ratio;_ *_SAPS:_* _Simplified Acute Physiology Score II. Level of significance ˂0.05._

References

1. Cella M, Chalder T. Measuring Fatigue in Clinical and Community Settings. Journal of Psychosomatic Research. 2010;69(1):17-22. http://dx.doi.org/https://doi.org/10.1016/j.jpsychores.2009.10.007.

2. Snaith RP. The Hospital Anxiety And Depression Scale. Health and Quality of Life Outcomes. 2003;1:29. http://dx.doi.org/10.1186/1477-7525-1-29.

3. Needham DM. Improving Long-Term Outcomes Research for Acute Respiratory Failure: The Outcomes After Critical Illness and Surgery (OACIS) Group at Johns Hopkins University School of Medicine; 2020 [Available from: https://www.improvelto.com/instruments/].

4. Mykletun A, Stordal E, Dahl AA. Hospital Anxiety and Depression (HAD) Scale: Factor Structure, Item Analyses and Internal Consistency in a Large Population. British Journal of Psychiatry. 2001;179(6):540-4. http://dx.doi.org/10.1192/bjp.179.6.540.

5. Hosey MM, Leoutsakos J-MS, Li X, Dinglas VD, Bienvenu OJ, Parker AM, et al. Screening for Posttraumatic Stress Disorder in ARDS Survivors: Validation of the Impact of Event Scale-6 (IES-6). Critical Care. 2019;23(1):276. http://dx.doi.org/10.1186/s13054-019-2553-z.

6. Thoresen S, Tambs K, Hussain A, Heir T, Johansen VA, Bisson JI. Brief Measure of Posttraumatic Stress Reactions: Impact of Event Scale-6. Social Psychiatry. 2010;45(3):405-12. http://dx.doi.org/10.1007/s00127-009-0073-x.

7. Eid J, Larsson G, Johnsen BH, Laberg JC, Bartone PT, Carlstedt B. Psychometric Properties of the Norwegian Impact of Event Scale-Revised in a Non-Clinical Sample. Nordic Journal of Psychiatry. 2009;63(5):426-32. http://dx.doi.org/10.1080/08039480903118190.

8. Cotes JE. Medical Reasearch Council Qestionnaire on Respiratory Symptoms (1986). The Lancet. 1987;330(8566):1028. http://dx.doi.org/https://doi.org/10.1016/S0140-6736(87)92593-1.
